# Burden of acute kidney injury and 90-day mortality in critically ill patients

**DOI:** 10.1186/s12882-019-1645-y

**Published:** 2019-12-31

**Authors:** Renske Wiersema, Ruben J. Eck, Mikko Haapio, Jacqueline Koeze, Meri Poukkanen, Frederik Keus, Iwan C. C. van der Horst, Ville Pettilä, Suvi T. Vaara

**Affiliations:** 1 Department of Critical Care, University of Groningen, University Medical Center Groningen, Groningen, The Netherlands; 2 Division of Intensive Care Medicine, Department of Anesthesiology, Intensive Care and Pain Medicine, University of Helsinki and Helsinki University Hospital, Helsinki, Finland; 3 Department of Internal medicine, University of Groningen, University Medical Center Groningen, Groningen, The Netherlands; 4 Nephrology, University of Helsinki and Helsinki University Hospital, Helsinki, Finland; 5 Department of Anaesthesia and Intensive Care, Lapland Central Hospital, Rovaniemi, Finland; 6 Department of Intensive Care, Maastricht University Medical Center+, Maastricht University, Maastricht, The Netherlands

**Keywords:** Acute kidney injury, Burden, Mortality, Critically ill, Prediction models

## Abstract

**Background:**

Mortality rates associated with acute kidney injury (AKI) vary among critically ill patients. Outcomes are often not corrected for severity or duration of AKI. Our objective was to analyse whether a new variable, AKI burden, would outperform 1) presence of AKI, 2) highest AKI stage, or 3) AKI duration in predicting 90-day mortality.

**Methods:**

Kidney Diseases: Improving Global Outcomes (KDIGO) criteria using creatinine, urine output and renal replacement therapy were used to diagnose AKI. AKI burden was defined as AKI stage multiplied with the number of days that each stage was present (maximum five), divided by the maximum possible score yielding a proportion. The AKI burden as a predictor of 90-day mortality was assessed in two independent cohorts (Finnish Acute Kidney Injury, FINNAKI and Simple Intensive Care Studies I, SICS-I) by comparing four multivariate logistic regression models that respectively incorporated either the presence of AKI, the highest AKI stage, the duration of AKI, or the AKI burden.

**Results:**

In the FINNAKI cohort 1096 of 2809 patients (39%) had AKI and 90-day mortality of the cohort was 23%. Median AKI burden was 0.17 (IQR 0.07–0.50), 1.0 being the maximum. The model including AKI burden (area under the receiver operator curve (AUROC) 0.78, 0.76–0.80) outperformed the models using AKI presence (AUROC 0.77, 0.75–0.79, *p* = 0.026) or AKI severity (AUROC 0.77, 0.75–0.79, *p* = 0.012), but not AKI duration (AUROC 0.77, 0.75–0.79, *p* = 0.06). In the SICS-I, 603 of 1075 patients (56%) had AKI and 90-day mortality was 28%. Median AKI burden was 0.19 (IQR 0.08–0.46). The model using AKI burden performed better (AUROC 0.77, 0.74–0.80) than the models using AKI presence (AUROC 0.75, 0.71–0.78, *p* = 0.001), AKI severity (AUROC 0.76, 0.72–0.79. *p* = 0.008) or AKI duration (AUROC 0.76, 0.73–0.79, *p* = 0.009).

**Conclusion:**

AKI burden, which appreciates both severity and duration of AKI, was superior to using only presence or the highest stage of AKI in predicting 90-day mortality. Using AKI burden or other more granular methods may be helpful in future epidemiological studies of AKI.

## Background

Acute kidney injury (AKI) is an abrupt decline in renal function which is defined by the Kidney Disease Improving Global Outcomes (KDIGO) criteria and based on changes in plasma creatinine (Cr), urine output, and use of renal replacement therapy (RRT) [1]. AKI has become a primary research focus within intensive care medicine [2] and many studies have focused on incidence, risk factors, and outcomes of AKI [3–5]. The mortality rates in patients with AKI range from 20 to 60%, most likely due to the heterogeneous populations and the variation in the use of AKI definitions [6].

Studies generally focus on the presence of AKI as a dichotomous variable or report the maximum stage, but often do not incorporate duration of AKI. Yet, both higher severity and longer duration of AKI are associated with increased hospital and long-term mortality [7–10]. Moreover, patients fulfilling both Cr and urine output criteria of AKI have been found to constantly have higher mortality compared to those with only one of the two criteria [7, 11]. Furthermore, the time-dependant nature of AKI and renal recovery has been shown to have an important prognostic impact [12, 13]. However, no study has incorporated the duration of different stages of AKI and evaluated their association with outcomes.

Theoretically, one would expect a prediction model including not only AKI severity but also AKI duration (in here referred to as “AKI burden”), to better separate patients according to their risk of death. For instance, a patient with transient AKI Stage 1 on day 2 of hospital admission would be expected to have a lower risk of death compared to a patient with AKI Stage 1 during the first 5 days of admission [8]. Classifying AKI patients according to their AKI burden compared to only AKI stage may partially explain heterogeneity in this patient group.

Our objective was to analyse whether a new variable, AKI burden, would predict 90-day mortality better than either 1) the presence of AKI, 2) the highest AKI stage, or 3) the duration of AKI alone, by conducting post-hoc analyses of two independent cohorts: the Finnish Acute Kidney Injury (FINNAKI) study [3], and the Simple Intensive Care Studies-I (SICS-I) [14]. We hypothesized that AKI burden would predict 90-day mortality better than the presence, the duration, or the severity of AKI only.

## Methods

### Study design, setting and participants

This study was a post-hoc analysis of the FINNAKI [3] and SICS-I cohort studies [14].

The FINNAKI was a prospective, observational, multicentre cohort study on the incidence, risk factors, and outcomes of AKI in 17 Finnish ICUs between 1 September 2011 and 1 February 2012. All emergency ICU admissions, regardless of the expected length of ICU stay, and all elective patients expected to stay in the ICU for more than 24 h were included. The excluded patients were: 1) patients under 18 years of age; 2) elective patients whose expected length of stay was less than 24 h; 3) readmitted patients who had received RRT during the previous ICU admission; 4) patients on chronic dialysis; 5) patients with insufficient language skills or not permanently living in Finland; 6) intermediate care patients; 7) transferred patients who had already participated in the study for 5 days; and 8) organ donors. In the current study, we further excluded patients from one study site as data of urine output were collected by different method. The Ethics Committee of the Department of Surgery in Helsinki University Central Hospital approved the FINNAKI study protocol with a deferred, written consent obtained from the patient or proxy as soon as possible. The Finnish National Institute of Health approved data collection from medical records of deceased patients. Statistics Finland provided data on 90-day survival status.

The SICS-I was a prospective observational single-centre cohort study on the association between physical examination and cardiac output conducted between 25 March 2015 and 4 July 2017 and included all acutely admitted critically ill patients in one ICU in the northern Netherlands [15]. Exclusion criteria were discharge within 24 h and/or absence of informed consent. The local ethical institutional review board approved this study.

### Data source, variables and study size

In both cohorts, daily AKI status was defined according to the complete KDIGO criteria based on plasma creatinine (Cr), hourly urinary output (all patients had urinary catheters) and the use of RRT separately [16]. Day 1 was defined as the calendar day of admission. The observation period was the first five calendar days. Baseline Cr in the FINNAKI database was defined as the most recent value from the previous year excluding the week preceding admission. If unavailable, baseline Cr was estimated using the Modification of Diet in Renal Disease (MDRD) equation as recommended [17]. In the SICS-I cohort an absolute baseline Cr was not available and was also estimated using the MDRD equation in all patients except when suffering chronic renal failure. Information about whether patients suffered from chronic renal failure with in the SICS-I cohort was available from the Nationale Intensive Care Evaluatie (NICE) registry, where a baseline serum creatinine above 177 μmol/L was defined as chronic renal failure [18]. In the FINNAKI cohort, the time labels of each UO recording along with the amount of urine and patient weight were transferred from the electronic patient data management systems to an electronic calculator maintained by Tieto Ltd., which provided the UO in mL/kg/h for all data. The study size was set by the sizes of the available cohorts. The AKI burden was considered as the main determinant in this analysis. The primary outcome was 90-day mortality, for which data on patients’ vital status were obtained from municipal record databases for FINNAKI (Finnish population register) and SICS-I.

### AKI burden

The AKI burden was calculated over a maximum of 5 days. We first assigned a different weight to each level of AKI (AKI 1 = 1, AKI 2 = 2, AKI 3 = 3), and we scored each weight twice daily, both for Cr and urine output. To account for the duration of AKI, we then multiplied the total weight with the observation time in days. Finally, we divided this score by the maximum possible score during the observation period to prevent underestimation of AKI burden in patients with missing data (the maximum score was 30, if there were no missing data, for examples see Table 1). The AKI burden at each specific day was maximal (i.e. 6) if patients received RRT. Higher proportions reflect higher AKI burden. We used arbitrarily defined cut-offs to create three categories of AKI burden (low burden, <0.25; medium burden, 0.25–0.50; high burden, >0.75). To ensure burden scores were representative, we evaluated mortality rates in different subtypes of AKI.

**Table 1:** Examples of AKI Burden calculations

	Stage Day 1		Stage Day 2		Stage Day 3		Stage Day 4		Stage Day 5		Available data	Maximal score	Actual score	Burden
Pt.	UO - Cr		UO - Cr		UO - Cr		UO - Cr		UO - Cr					
1	2	3	3	3	.	.	.	.	.	.	4	12	11	11/12 = 0.92
2	.	.	1	1	0	0	0	0	0	0	8	24	2	2/24 = 0.08
3	0	0	0	0	0	0	0	0	0	0	10	30	0	0/30 = 0
4	2	2	3	3	3	3	3	3	3	3	10	30	28	28/30 = 0.93
5	1	1	3	3	3	3	.	.	.	.	6	18	14	14/18 = 0.77
RRT*														

Abbreviations: *Pt* Patient, *UO* Urine Output, *Cr* Creatinine, *RRT* Renal replacement therapy. Maximal score = days multiplied by 3 (highest stage of AKI). *Although based on UO and Cr, the patient had maximal stage 2, as the patient received RRT, the burden for that day was maximal, so 6

### Missing data

We handled missing data in each cohort following the original statistical methods. In the FINNAKI cohort no imputations were performed. In the SICS-I cohort, predictor variables were imputed using multiple imputations, but data on Cr, urine output, and mortality were not imputed [19]. We appreciated missing Cr and urine output data by only calculating the AKI burden as a proportion of data which were available (i.e., neglecting missing data) (Table 1). Further, we performed a sensitivity analysis excluding patients who deceased during the five-day observation period to assess whether this influenced the models.

### Statistical analysis

We present data as means (with standard deviations (SD)) or medians (with interquartile ranges (IQR)) depending on distributions. Categorical data are presented in proportions. Outcomes are calculated as odds ratios (OR) with 95% confidence intervals (CI). Student’s T-test, Mann-Whitney U test or the Chi-square tests are used as appropriate.

We used the previously published logistic regression model from FINNAKI and the logistic regression model of SICS-I as the main models for predicting 90-day mortality; these models will from now on be referred to as the ‘original models’. The original model of FINNAKI included age, presence of chronic liver failure, malignancy, arteriosclerosis, diabetes mellitus, systolic heart failure, or chronic immunosuppression, pre-morbid functional status (regarding daily activities), as well as presence of hypotension or resuscitation prior to ICU admission and ICU admission type [20]. The original model of SICS-I included age, vasopressor dose, respiratory rate, atrial fibrillation, systolic and diastolic blood pressure, level of consciousness following the alert, verbal, pain or unresponsive (AVPU) score, central temperature, and mottling rate scored on the knee (obtained during a one-time examination within 24 h of admission) [19].

In each cohort, we constructed four models: the original model including presence of AKI as a dichotomous variable (model A), the original model including the highest stage of AKI as severity of AKI (model B), the original model and the duration of AKI (model C) and the original model and the AKI burden (model D). We calculated pseudo R^2^, applied Hosmer-Lemeshow goodness of fit test, assessed area under the ROC and used DeLongs test to compare the area under the ROC of the models. *P*-values of <0.05 were considered statistically significant.

## Results

### FINNAKI - Patients

Of 2901 FINNAKI study patients, 92 patients were excluded due to difference in recording urine output at one study site. In that study site, urine output was collected cumulatively instead of hourly, which made it not possible to assess the hourly urine output following the method used for the FINNAKI study. The median observation period was 4 days (IQR 2–5); 1601 patients (55%) were discharged, and 167 patients (6%) deceased before 5 days.

### FINNAKI - AKI

Of the remaining 2809 patients, 1096 patients (39%) had AKI at least once during the first 5 days of ICU admission (Additional file 1: Figure S1). AKI Burden could be calculated for 2793 patients (99%). The proportions of missing values for Cr and urine output during the study period are shown in Additional file 1: Table S1. Among 1096 patients with AKI during ICU stay the median AKI burden observed during the first 5 days of admission was 0.17 (IQR 0.07–0.50) (Additional file 1: Figure S2). Of these, 641 patients (58%) had low burden (< 0.25), 186 patients (17%) had medium burden (0.25–0.50) and 269 patients (25%) had high burden (> 0.50). The highest stage of AKI was stage 1 in 482 patients (44%), stage 2 in 224 patients (20%), and stage 3 in 390 patients (36%). RRT was instigated in 260 patients (24%).

### FINNAKI - Outcomes

**Fig. 1:**
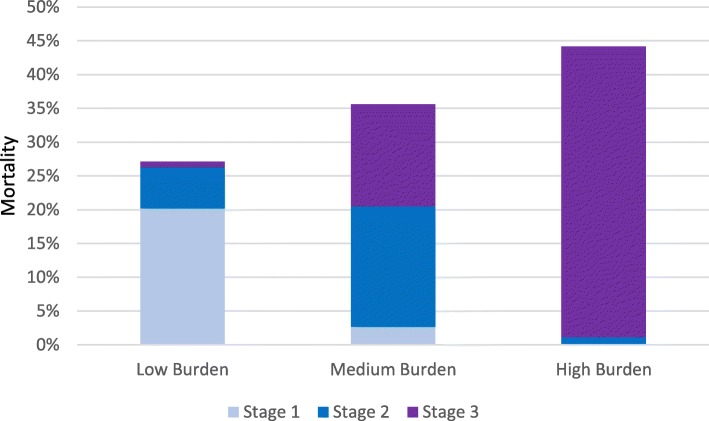
Burden of acute kidney injury and subsequent mortality rate in the FINNAKI cohort. * Low burden = below 0.25, medium = above 0.25 but below 0.50, high = above 0.50

At 90-day follow up, 653 patients (23%) had died. Table 2 presents baseline characteristics of survivors and non-survivors. In patients with low, medium and high AKI burden, mortality rates were 27% (CI 23–31), 35% (CI 29–42) and 44% (CI 38–50), respectively (Fig. 1). Univariate logistic regression showed that presence of AKI, the severity of AKI, the duration of AKI, and AKI burden all were associated with 90-day mortality (Table 3). There was no significant difference in mortality between patients who had AKI stage 1 based on urine output and patients who had AKI stage 1 based on Cr (*p* = 0.88).

**Table 2:** Baseline characteristics of included patients from the FINNAKI cohort

	Data available	Survivors *N* = 2156	Data available	Non-survivors *N* = 653
Age, years (SD)	2156	58.9 (16.8)	653	67.9 (14.0)
Gender, male (%)	2156	1368 (63.5)	653	424 (64.9)
BMI, kg/m2 (SD)	2137	29.1 (27.7)	647	27.9 (16.5)
Baseline creatinine, mmol/L (IQR)	1340	74 (60–90)	450	78 (63–103)
Co-morbidities				
Hypertension, n (%)	2143	979 (45.7)	650	350 (53.8)
Diabetes mellitus, n (%)	2156	470 (21.8)	653	137 (21.0)
COPD, n (%)	2142	180 (8.4)	650	73 (11.2)
Systolic heart failure, n (%)	2138	213 (10.0)	647	109 (16.8)
Chronic liver failure, n (%)	2136	60 (2.8)	643	49 (7.6)
Chronic renal failure*, n (%)	2147	118 (5.5)	650	63 (9.7)
Pre-ICU daily medication				
Diuretic, n (%)	2125	550 (25.9)	634	229 (36.1)
Statin, n (%)	2127	633 (29.8)	639	195 (30.5)
Metformin, n (%)	2125	270 (12.7)	639	65 (10.2)
NSAID, n (%)	2080	190 (9.1)	618	51 (8.3)
Corticosteroid, n (%)	2132	118 (5.5)	642	96 (15.0)
Admission				
Emergency, n (%)	2156	1828 (85.9)	653	623 (95.7)
Operative, n (%)	2156	824 (38.2)	653	164 (25.1)
Mechanical ventilation, n (%)	2156	1404 (65.1)	653	531 (81.3)
Vasopressor use, n (%)	2156	1212 (56.2)	653	461 (70.6)
SAPS II (SD)	2156	35.2 (14.0)	653	54.4 (18.2)
SOFA score (SD)	2156	6.4 (3.1)	653	9.6 (3.9)

*Chronic renal failure defined as eGRF <60 ml/min/1.73 m2. Abbreviations: *SD* Standard Deviation, *BMI* Body Mass Index, *IQR* Inter Quartile Range, *COPD* Chronic Obstructive Pulmonary Disease, *NSAID* Non-steroidal anti- inflammatory drugs, *SAPS II* Simplified Acute Physiology Score II, *SOFA* Sequential Organ Failure Assessment score

**Table 3:** Multivariate models in FINNAKI

	Univariate OR^a^ (95% CI)	Multivariate OR^a^ (95% CI)	Pseudo R^2^	H-L GoF	*p*-value H-L	AUROC	95% CI
Original model			0.15	8.22	0.41	0.76	0.74–0.78
(A) Presence of AKI^b^	2.35 (1.96–2.81)	1.94 (1.59–2.37)	0.16	8.54	0.38	0.77	0.75–0.79
(B) Severity of AKI^b^	1.45 (1.35–1.57)	1.37 (1.25–1.49)	0.16	7.26	0.51	0.77	0.75–0.79
(C) Duration of AKI^b^	3.46 (2.74–4.37)	2.74 (2.10–3.57)	0.16	5.93	0.65	0.77	0.75–0.79
(D) AKI burden^b^	4.97 (3.65–6.77)	4.56 (3.22–6.53)	0.17	10.71	0.22	0.78	0.76–0.80

Abbreviations: *AKI* Acute Kidney Injury, *OR* Odds Ratio, *CI* Confidence Interval, *H-L* Hosmer Lemeshow, *GoF* Goodness of Fit, *AUROC* Area under the receiver operating curve.^a^OR of the different methods for AKI in the corresponding model, which were categorical (A and B), or per additional day or point (C and D). ^b^Model A, B and C included 2732 patients, Model D included 2707 patients

### FINNAKI - multivariate models

The Odds Ratio (OR) for 90-day mortality was the highest for AKI burden 4.56 (95%CI 3.22–6.53). Discrimination (AUC) of the burden model (AUROC 0.78, 0.76–0.80) was statistically significantly superior compared to presence of AKI (AUROC 0.77, 0.75–0.79, *p* = 0.023), severity of AKI (AUROC 0.77, 0.75–0.79, *p* = 0.015), but not statistically significantly different compared to the duration of AKI (AUROC 0.77, 0.75–0.79, *p* = 0.06) (Table 3). The model with duration of AKI did not have a statistically significant better performance compared to the presence of AKI (*p* = 0.08) or the severity of AKI (*p* = 0.63). The sensitivity analysis excluding patients deceased within 5 days confirmed these results.

### SICS-I

The SICS-I cohort included 1075 patients. The median observation period was 3 days (IQR 2–5); 575 patients (53%) were discharged and 118 patients (11%) deceased within 5 days. In total, 603 patients (56%) had AKI at some point during the first 5 days of their admission. AKI burden could be calculated in 1055 patients (98%) and median burden of all 603 patients with AKI was 0.19 (IQR 0.08–0.46) (Additional file 1: Figure S3). The proportions of missing values for Cr and urine output during the study period are shown in Additional file 1: Table S2. Of the 1075, 297 patients (28%) had died during 90-day follow-up, which was significantly associated with mortality (Additional file 1: Figure S4). The observed OR was 6.03 (95%CI 3.50–10.38) for AKI burden. The AUROC of the model including AKI burden (0.77, 95%CI 0.74–0.80) was better compared to the AUROC of the models including the presence of AKI (0.75, 95%CI 0.71–0.77) (*p* = 0.001), the severity of AKI (0.76, 95%CI 0.72–0.79) (*p* = 0.008) or the duration of AKI (0.76, 95%CI 0.73–0.79) (*p* = 0.009) (Table 4).

**Table 4:** Multivariate models in SICS-I

	Univariate OR^a^ (95% CI)	Multivariate OR^a^ (95% CI)	Pseudo R^2^	H-L GoF	*p*-value H-L	AUROC	95% CI
Original model			0.15	8.22	0.41	0.76	0.74–0.78
(A) Presence of AKI^b^	2.12 (1.60–2.82)	1.72 (1.24–2.36)	0.14	2.87	0.94	0.75	0.71–0.78
(B) Severity of AKI^b^	1.44 (1.29–1.61)	1.36 (1.20–1.54)	0.15	6.53	0.58	0.76	0.72–0.79
(C) Duration of AKI^b^	3.13 (2.26–4.32)	2.68 (1.84–3.92)	0.15	3.62	0.88	0.76	0.73–0.79
(D) AKI burden^b^	6.95 (4.36–11.11)	6.03 (3.50–10.38)	0.16	8.18	0.42	0.77	0.74–0.80

Abbreviations: *AKI* Acute Kidney Injury, *OR* Odds Ratio, *CI* Confidence Interval, *H-L* Hosmer Lemeshow, *GoF* Goodness of Fit, *AUROC* Area under the receiver operating curve. ^a^OR of the different methods for AKI in the corresponding model, which were categorical (A and B), or per additional day or point (C and D). ^b^All models included 1032 patients

## Discussion

### Key results

In this post-hoc analysis of two large prospective cohorts we found that AKI burden was superior for prediction of 90-day mortality in comparison to severity or presence of AKI. In comparison to duration of AKI, 90-day mortality prediction was improved by AKI burden in the SICS-I cohort, but remained comparable in the FINNAKI cohort.

### Comparison to previous studies

Our results corroborate the findings of a study by Mandelbaum et al. [8], who investigated the empirical relationships between oliguria, Cr disturbances, and mortality. However, that study was a single centre study and did not use a fixed mortality endpoint. Coca et al. investigated both duration and severity of post-operative AKI separately, and showed similar results to ours in diabetic patients [9]. A study by Truche et al. aimed to investigate the association of AKI duration with mortality and found that both the duration of AKI and the duration of renal recovery were associated with 28-day mortality [12]. They argued that time-dependent variables representing the course of AKI should be taken in to account for diagnostic and prognostic purposes, however, no urine output data were available and thus these conclusions were based on Cr AKI only [12]. We confirmed these conclusions in two separate cohorts, where both Cr and urine output were available, and additionally incorporated severity to establish the AKI burden.

### Implications

Data of incidence, staging and mortality of AKI among critically ill patients are increasing [21]. Many studies focus on prediction models for mortality and on finding appropriate ways for stratification of AKI in these models. Modelling AKI appeared difficult due to different AKI criteria and more importantly, different types of AKI exist. There is, however, not yet one superior method for integrating the different stages of AKI, varying from a stage 1 AKI based on urine output to a stage 3 AKI based on Cr, and duration of AKI. AKI burden as a sort of area under the curve to represent the severity or impact of AKI in mortality models may be a step toward for including AKI in prediction models. AKI burden can be calculated easily, handles missing data and could potentially incorporate duration along with severity. We showed, in two independent large cohorts that AKI burden helps to better appreciate the severity and duration of different types of AKI. The two cohorts differed in terms of selection criteria and logically also in AKI incidences and mortality rates. More importantly, the original models (FINNAKI admission model based on previous medical history versus SICS model based on signs of clinical examination) were very different. As our hypothesis stands for both cohorts, AKI burden improved the prognostic performance of the 90-day mortality model, irrespective of the differences in selection criteria and the admission variables which formed the model.

### Limitations

There are some limitations that need to be considered. First, as our data sets were to some extent incomplete, we analysed the available data to censor and correct for missing data as much as possible. Nevertheless, we were lacking data on Cr and urine output all 5days in both cohorts, as patients could have been discharged to the ward (in which case a low burden would be expected) or deceased during these first 5 days (in which case a higher burden would be expected). However, we observed no change in the models after excluding the patients who died during the five-day observation period. Although missing data are handled to some extent by AKI burden, those may still have influence: in a case with few valid values close to 0 and missing data, AKI burden may be underestimated; while in a case with few valid values close to 3 and missing data, it may be overestimated. Despite these shortcomings AKI burden still was a statistically stronger predictor than AKI presence and severity. Second, ideally, the observation period to estimate AKI burden would be longer, e.g. 7 days [22]. The observation period of 5 days were calendar days, meaning that the observation time is not entirely equal throughout all patients, although we corrected for missing data in the burden calculation. Optimally, the AKI burden would be a proportion of the same amount of data for every patient. Unfortunately, this remains a challenge for observational studies in critical care, as length of stay varies between patients and urine output data may be missing while transferred to the ward. Using multiple imputations could have optimized our analysis. Third, we used the MDRD formula to estimate baseline creatinine if not available. This method has inherent limitations as it may underestimate AKI in younger patients and overestimate AKI in the elderly [23]. Fourth, our approach was to give equal weight to both urine output and Cr AKI stages, although some reports have illustrated that different stages of urine output and Cr AKI handled separately do not associate with mortality with equal strength [7]. However, sensitivity analysis, showed results to be robust as there was no significant difference in mortality between AKI stage 1 based on urine output or Cr. Finally, we anticipated that AKI burden had resulted in a more clinically significant increase in the prediction ability compared to presence or severity of AKI. Despite the statistically significant difference, the clinical significance of this finding may be limited. However, we believe that this more granular method is helpful in future epidemiological research.

### Generalizability

We included a heterogeneous population; investigation of subgroups might show our burden model performs better or worse in predicting mortality in patient groups with different AKI etiologies, for instance sepsis or shock patients. We validated our results in a separate cohort, which is a major strength of our study and shows that our results apply to different cohorts. Our observations encourage researchers to look beyond stage or duration of AKI and incorporate AKI burden as risk factor.

## Conclusions

We found that calculated AKI burden, which included both severity and duration of AKI, was superior compared to only the presence of AKI, or the severity of AKI for predicting 90-day mortality in two large, independent cohorts of critically ill patients. Using AKI burden or other more granular methods may be helpful in future epidemiological studies of AKI.

## Supplementary information

**Additional file 1: Table S1.** Percentages of missing data in FINNAKI. **Table S2.** Percentages of missing data in SICS-I. **Figure S1.** Flowchart of study inclusion in FINNAKI. **Figure S2.** Histogram presenting the burden of acute kidney injury in FINNAKI. **Figure S3.** Histogram presenting the burden of acute kidney injury in SICS-I. **Figure S4.** Burden of acute kidney injury and subsequent mortality rate in the SICS-I cohort.

## Data Availability

The datasets used during the current study are available from the corresponding author on reasonable request.

## Abbreviations

AKI: Acute kidney injury
AUROC: Area Under the Receiver Operating Curve
AVPU: Alert, Verbal, Pain, Unresponsive score
CI: Confidence interval
Cr: Creatinine
ICU: Intensive Care Unit
IQR: Inter Quartile Range
KDIGO: Kidney Disease Improving Global Outcome
MDRD: Modification of Diet in Renal Disease
OR: Odds Ratio
RRT: Renal replacement therapy
SD: Standard Deviation
