# Supplementary material for: Burden of acute kidney injury and 90-day mortality in critically ill patients
Source: BMC Nephrol. 2019 Dec 31;21:1. doi: 10.1186/s12882-019-1645-y (PMC6938017; doi:10.1186/s12882-019-1645-y)

**ELECTRONIC SUPPLEMENTAL MATERIAL – AKI Burden, Wiersema et al.**

| N = 2809 | Day 1* | Day 2 | Day 3 | Day 4 | Day 5 |
| --- | --- | --- | --- | --- | --- |
| Creatinine data missing | 1588 (57%) | 437 (15%) | 1069 (38%) | 1567 (56%) | 1873 (66%) |
| Urine output data missing | 179 (6%) | 183 (7%) | 862 (31%) | 1407 (50%) | 1752 (62%) |

**E-Table 1.** Missing data per observation day FINNAKI

* In the FINNAKI study, creatinine measured on ICU was registered. Many patients had pre-ICU creatinine values obtained for clinical use (i.e. on the emergency department), and therefore often no new creatinine was measured on ICU day 1. In this analysis they therefore could not be classified purely on calendar day 1.

| N = 1075 | Day 1* | Day 2 | Day 3 | Day 4 | Day 5 |
| --- | --- | --- | --- | --- | --- |
| Creatinine data missing | 5 (<0.01%) | 23 (2%) | 90 (8%) | 208 (19%) | 337 (31%) |
| Urine output data missing | 235 (22%) | 263 (24%) | 452 (42%) | 650 (60%) | 770 (72%) |

**E-Table 2.** Missing data per observation day SICS-I

* In the SICS-I study, creatinine measured either on the regular ward or on the ICU was registered.

**E-Figure 1.** Flowchart of study inclusion FINNAKI


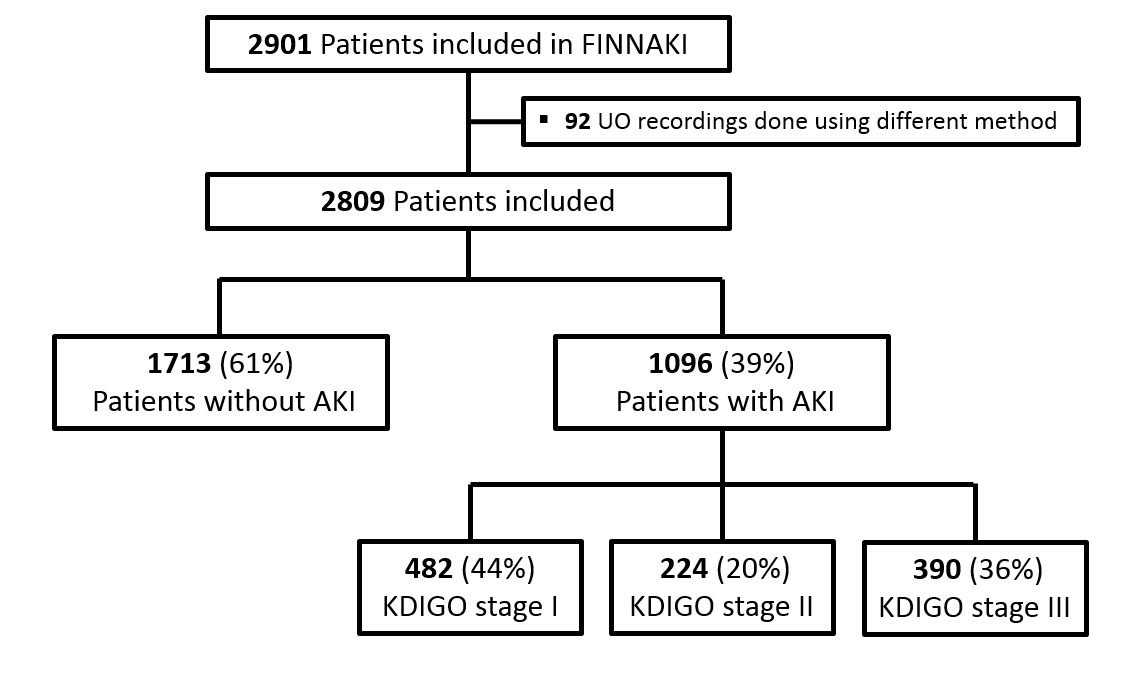


**E-Figure 2.** Histogram burden FINNAKI


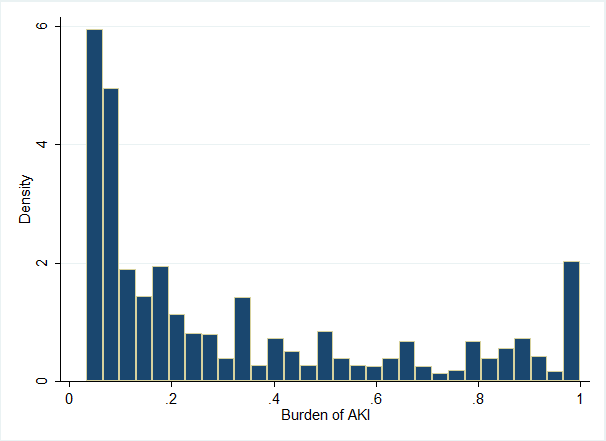


**E-Figure 3.** Histogram burden SICS-I


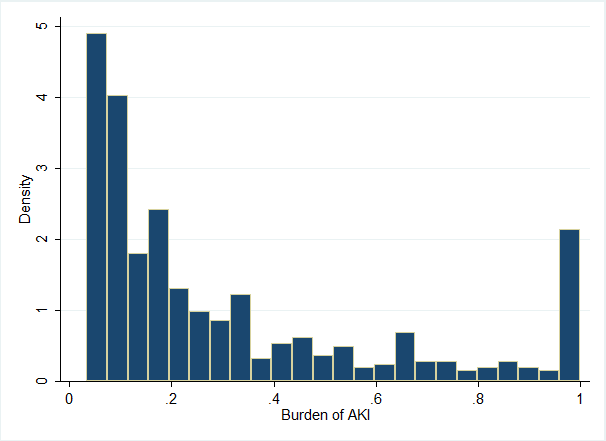


**E-Figure 4.** Burden of acute kidney injury and subsequent mortality rate (percentage) in the SICS-I cohort


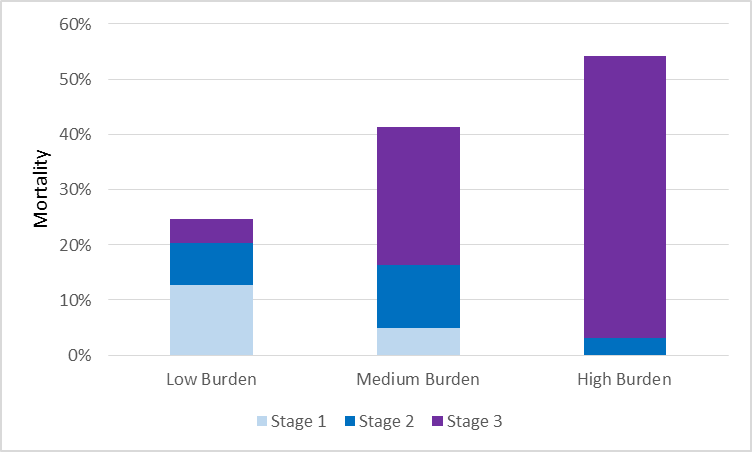

Supplement: Supplementary file 1 — Additional file 1: Table S1. Percentages of missing data in FINNAKI. Table S2. Percentages of missing data in SICS-I. Figure S1. Flowchart of study inclusion in FINNAKI. Figure S2. Histogram presenting the burden of acute kidney injury in FINNAKI. Figure S3. Histogram presenting the burden of acute kidney injury in SICS-I. Figure S4. Burden of acute kidney injury and subsequent mortality rate in the SICS-I cohort. [file 12882_2019_1645_MOESM1_ESM.docx]
